# Brachioradialis Involvement in Hirayama's Disease: An Atypical Presentation of a Rare Cervical Myelopathy

**DOI:** 10.7759/cureus.49354

**Published:** 2023-11-24

**Authors:** Aishwarya Malode, Naman Modi, Arihant Seth, Rishikesh Kamble, Ajay Mathur

**Affiliations:** 1 Internal Medicine, Sawai Man Singh Medical College, Jaipur, IND

**Keywords:** brachioradialis involvement, non-progressive amyotrophy, myelomalacia, cervical myelopathy, juvenile monomelic amyotrophy, hirayama's disease

## Abstract

Juvenile monomelic amyotrophy (JMA), also known as Hirayama's disease, is a rare cervical myelopathy that predominantly affects young Asian males. It is characterized by degeneration of anterior horn cells due to compression by the redundant dural sac. This study presents an atypical case of a 23-year-old Indian male who exhibited uncommon symptoms of JMA. The patient displayed progressive weakness and atrophy in the left forearm, including the usually spared brachioradialis muscle. Electrophysiological tests and MRI scans solidified the diagnosis of Hirayama's disease. After wearing a cervical collar for one year, the patient's condition stabilized, reinforcing the diagnosis. Unlike most JMA cases, this instance highlights the involvement of the brachioradialis muscle, underlining the variability in JMA presentations. A precise diagnosis is contingent upon clinical criteria, dynamic MRI, and electrophysiological findings. Recognizing these variations is crucial for early detection and appropriate management of the disease.

## Introduction

Juvenile monomelic amyotrophy (JMA) is a distinctive variant of cervical myelopathy, also known as Hirayama’s disease, juvenile non-progressive amyotrophy, and Soube’s disease [1]. The condition was first described by K. Hirayama in 1959 [1-6]. Predominantly, this disorder affects young Asian males with a male-to-female ratio ranging from 7:1 to 20:1 [2]. Typically, the onset of the disease is observed between the ages of 10 and 20, with the most pronounced presentation occurring between 20 and 30 years. This is succeeded by a plateau phase. Distinctly, the progression of the disease does not extend beyond five years [1-6], setting it apart from other anterior horn cell diseases. Although the exact pathophysiology remains elusive, the prevailing theory suggests a discordant growth between the vertebral column and its contents. This leads to an indentation of the cervical spinal cord by the posterior dural sac and the dural venous plexus during neck flexion. As a result, repeated micro-ischemia and anterior horn cell atrophy are induced [1-6]. This mechanistic explanation aligns with the disease's onset during the growth spurt phase, which later plateaus. A hallmark of this condition is it's presentation-typically involving unilateral or bilateral weakness and atrophy of distal muscles of the upper limbs, sparing the brachioradialis and showcasing distinctive MRI findings. It is a diagnosis of exclusion, and Toshiro’s criteria aid in this diagnosis [6].

## Case presentation

**Figure 1:**
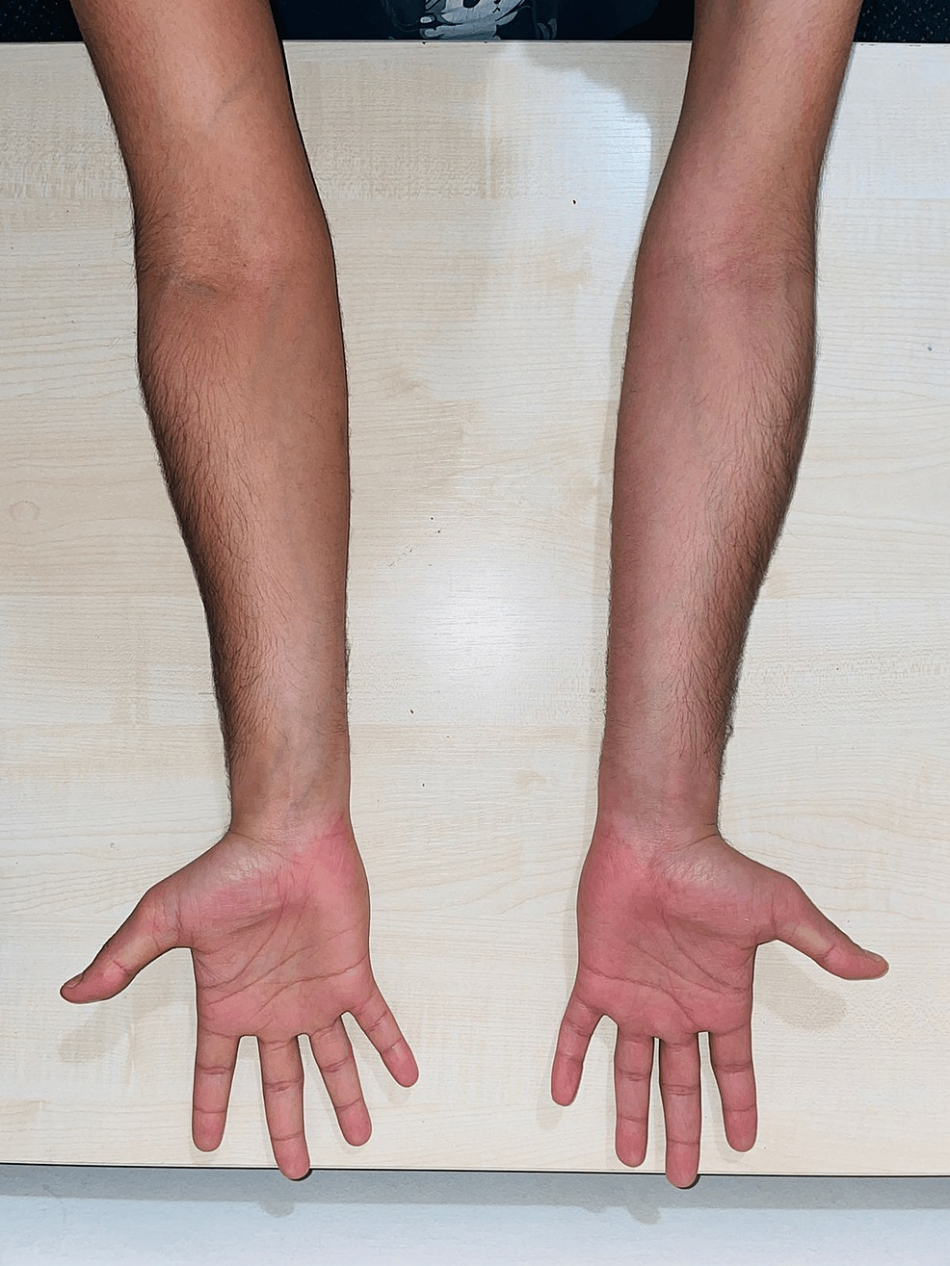
Image showing atrophy of the left forearm of the patient

**Figure 2:**
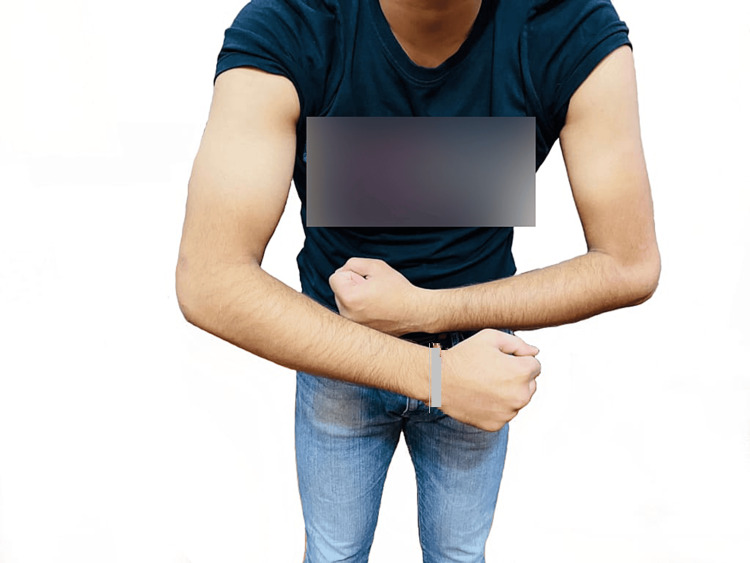
Image showing atrophied left upper limb of the patient

We report a case involving a 23-year-old male of Indian origin who presented with an insidious onset of progressively worsening weakness and thinning in his left forearm. His symptoms became noticeable over the past four years, particularly when he experienced difficulty lifting weights in the gym and dropped plates from his hand. Though hand movements were not compromised, a steadily increasing weakness in the grip strength of his left hand was evident. This progression lasted for three years and subsequently stabilized. A comprehensive neurological evaluation revealed that his higher mental functions, cranial nerves, sensory examination, and cerebellar function were all within normal limits. However, motor examination showed a noticeable reduction in muscle bulk in the left forearm (Figures 1-2), a power rating of four out of five in both the extensors and flexors of the elbow and wrist joint, atrophy and weakness in the brachioradialis muscle, and weakness in the dorsal and palmar interossei. Despite this, the power in the thumb abductors was preserved, and there was no apparent wasting of the small hand muscles. Deep tendon reflexes remained unchanged.

Routine laboratory examinations, including complete blood count (CBC), renal function test (RFT), liver function test (LFT), thyroid function test (TFT), erythrocyte sedimentation rate (ESR), C-reactive protein (CRP), and creatine phosphokinase-N-acetyl cysteine (CPK-NAC), all yielded results within the normal range. Tests for vasculitis, including antinuclear antibodies (ANA), ESR, and CRP, came back negative. A nerve conduction study (NCS) (Tables 1-3) revealed a decreased compound muscle action potential in the distribution of the left musculocutaneous nerve, showing a decrement of over 50% when compared to the right side. Despite this, distal latency and conduction velocity were found to be normal.

**Table 1:** Sensory nerve conduction study (NCS) data LAT - Latency, AMP - Amplitude, CV - Conduction Velocity.

	Left LAT (ms)	Left AMP (uV)	Left CV (m/s)
General Nerve Sensory			
Digit 1 - Median Wrist	1.67	12.5	72.5
Digit 3 - Median Wrist	2	16.1	70.5
Digit 5 - Ulnar Wrist	1.89	12.6	64
Lateral Cutaneous nerve			
Elbow, Forearm	1.67	15.6	72.5
Medial Cutaneous nerve			
Forearm, Elbow	1.92	16.2	78.6

**Table 2:** Motor nerve conduction study (NCS) data APB - Abductor Pollicis Brevis, ADM - Abductor Digiti Minimi.

	Left LAT (ms)	Left AMP (uV)	Left CV (m/s)
Median ABP Motor			
Wrist - ABP	3.13	17.2	
Elbow - Wrist	7.19	17.1	64.5
Ulnar ADM motor			
Wrist - ADM	2.25	21.5	
Below Elbow - Wrist	6.35	21.6	59.8
Above Elbow - Below Elbow	7.42	21.5	70.1

**Table 3:** F Wave response data.

	Left Shortest F-lat	Left persistence %
Median APB F-Response		
Wrist - APB	27.6	100
Ulnar ADM F-Response		
Wrist - ADM	26.9	100

The sensory nerve action potentials for bilateral median, ulnar, radial, axillary, and right musculocutaneous nerve were also normal. Electromyography (EMG) test results (Table 4) indicated fasciculations and reduced interference patterns in the musculocutaneous nerve distribution. 

**Table 4:** EMG Findings (30G/26G concentric needle electrode) EMG - Electromyography, Lt - Left, Rt - Right, PSW - Positive Sharp Waves, Fibs - Fibrillation Potential, IP - Interference Pattern.

Muscle	Spontaneous Activity			Voluntary Activity		
	Fibs/PSW	Fasciculations	Amp	Duration	Polyphasia	IP
Lt Deltoid	nil	++	Mild increase	Mild increase	Moderate increse	Moderate reduction
Rt Deltoid	nil	-	Normal	Normal	Normal	Full
Lt Extensor Digitorum	nil	-	Normal	Normal	Normal	Full
Rt Extensor Digitorum	nil	-	Normal	Normal	Normal	Full
Lt Extensor Indicis	nil	-	Normal	Normal	Normal	Full
Rt Extensor Indices	nil	-	Normal	Normal	Normal	Full
Lt Brachioradialis	nil	++	Moderate increase	Mild increase	Moderate reduction	Moderate reduction
Rt Brachioradialis	nil	-	Normal	Normal	Normal	Full
Lt Flexor Policis Longus	nil	-	Normal	Normal	Normal	Full
Rt Extensor Policis Longus	nil	-	Normal	Normal	Normal	Full
Lt Iliopsoas	nil	-	Normal	Normal	Normal	Full
Rt Iliopsoas	nil	-	Normal	Normal	Normal	Full
Lt Tibialis Anterior	nil	-	Normal	Normal	Normal	Full
Rt Tibialis Anterior	nil	-	Normal	Normal	Normal	Full
Lt Trapezius	nil	-	Normal	Normal	Normal	Full
Rt Trapezius	nil	-	Normal	Normal	Normal	Full
Lt Triceps	nil	-	Normal	Normal	Normal	Full
Rt Triceps	nil	-	Normal	Normal	Normal	Full
Lt Vastus Medialis	nil	-	Normal	Normal	Normal	Full
Rt Vastus Medialis	nil	-	Normal	Normal	Normal	Full
Lt Biceps	nil	++	Mild increase	Mild increase	Moderate increase	Moderate reduction
Rt Biceps	nil	-	Normal	Normal	Normal	Full

**Figure 3:**
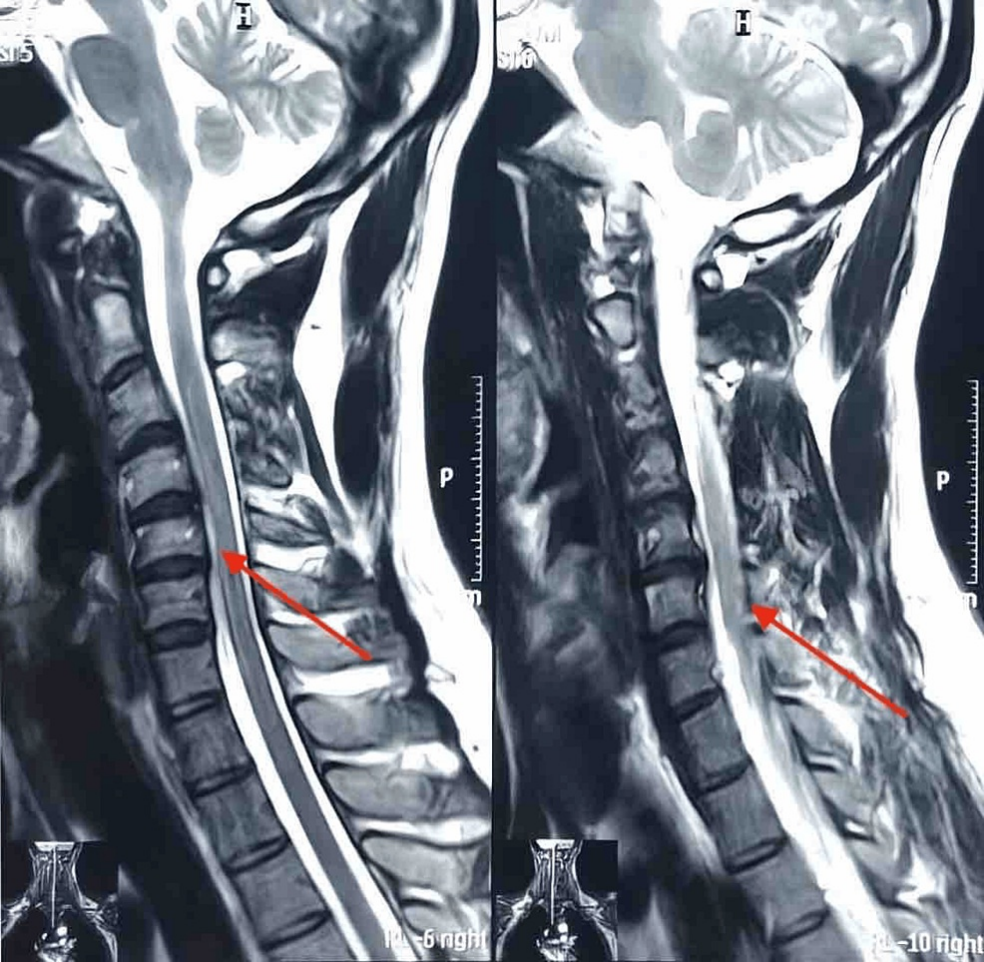
T2 MRI sequence showing cervical myelomalacia

An MRI of the cervical spine revealed cervical myelomalacia (Figure 3), accompanied by asymmetric cord atrophy (L>R) in a neutral position. Dynamic MRI taken during neck flexion suggested anterior shifting of the posterior dural sac, leading to subsequent cervical cord compression. The diagnosis of Hirayama’s disease was established based on the cumulative clinical, electrophysiological, and MRI findings [1-6]. The patient was advised to wear a cervical collar and avoid hyperflexing the neck. The disease showed no progression over the subsequent year of follow-up, further supporting our diagnosis.

## Discussion

Hirayama’s disease is recognized as a benign form of non-progressive compressive cervical myelopathy leading to asymmetrical weakness and wasting of the upper limb muscles due to the involvement of myotomes spanning the C5-T1 segment, predominantly those of the C7-T1 segments [1-6]. Although there have been familial presentations, ascribed to mutations in the KIAA1377 and C5ORF42 genes [6], the disease is typically sporadic, as seen in the discussed case [1]. It is postulated that a combination of environmental factors, ethnicity, cultural practices, and behavioral habits contribute to susceptibility to the disease [6].

The exact etiopathogenesis of Hirayama’s disease remains elusive. However, the most accepted theory is the degeneration of anterior horn cells in the cervical segment, caused by micro-ischemia resulting from compression and indentation by the posterior dural sac. This displacement is believed to arise from increased laxity of the cervical dura mater anchored at C2-C3 segments, leading to forward movement during neck flexion [1-6], thus causing micro-trauma and micro-ischemia of the anterior horn cells.

The disease is a type of focal amyotrophy that frequently affects one side, with a higher incidence on the left than the right, as in our case. In approximately 10% of cases, it may manifest bilaterally, either symmetrically or asymmetrically [1]. Typically, the brachioradialis muscle is spared, leading to the characteristic "Oblique amyotrophy" [2,6]. However, in the case we presented, there was evident involvement of the brachioradialis muscle, in conjunction with forearm muscles, and relative sparing of small hand muscles, rendering our case particularly unusual. Most commonly, the weakness is localized in the C7-T1 myotome, leading to pronounced distal muscle weakness and atrophy. In our patient, there was weakness across multiple functions: elbow flexion and extension, as well as, various forearm and hand movements, leading to difficulties in grasping objects with the left hand. Notably, weakness in elbow flexion and extension is uncommon. Some patients may exhibit aggravated symptoms with neck flexion and cold exposure, known as "cold paresis," a phenomenon absent in our case. Up to 30% of Hirayama’s disease patients might display low-amplitude tremors [1]. Toshiro’s criteria are often utilized to clinically suspect the diagnosis of Hirayama’s disease [6].

Diagnostic confirmation often relies on MRI and electrophysiological studies. A routine MRI of the cervical spine may either appear normal or might show non-specific myelomalacia in the lower cervical segments [1-7]. Often, myelomalacia can be asymmetric, predominantly affecting the left side, as in our patient, with axial T2 images illustrating the "snake-eye" appearance [1]. A dynamic MRI, with the neck flexed at an angle of 30-40 degrees, is essential for diagnosis, often revealing an anterior shift of the posterior dura, enlargement of the posterior dural sac, and cord compression [1-7]. These observations are consistent with our case. Nerve conduction studies (NCS) can sometimes illustrate lower ulnar nerve compound muscle action potentials (CAMPs) compared to the median and radial nerve. Our patient’s NCS indicated a low CAMP in the distribution of the left musculocutaneous nerve, which is not a usual finding but had a normal conduction velocity and sensory modalities, in line with our case [1-5].

The differential diagnosis for Hirayama’s disease should take into account conditions such as amyotrophic lateral sclerosis, spinal muscular atrophy, C8-T1 radiculopathy, syringomyelia, cervical spondylotic myelopathy, post-polio syndrome, and toxic neuropathy [6].

Hirayama’s disease is generally self-limiting, with 95% of patients seeing a plateau in symptoms after five years [2,6]. Most patients, like ours, respond favorably to conservative management using a cervical collar. In the year following his diagnosis, our patient neither improved nor deteriorated. Surgical options, such as cervical decompression combined with fusion duroplasty, have also been explored for treating the disease [1-6].

## Conclusions

Hirayama’s disease, while a comparatively rare entity, stands as a crucial differential diagnosis, especially in young Asian male patients who exhibit asymmetrical upper limb weakness without any notable medical antecedents. Given its potential for varied and atypical presentations, it becomes imperative for clinicians to employ a multifaceted diagnostic approach. Integrating dynamic MRI results, electrophysiological findings, and rigorous clinical criteria ensures a comprehensive and accurate identification of the condition. Recognizing and diagnosing Hirayama’s disease early can guide appropriate management strategies, potentially altering the course and improving patient outcomes.
